# Cross-Verification and Consistency Evaluation of Monochromator-Based Radiance Calibration Systems Under Varying Bandwidths Against an SI-Traceable Reference

**DOI:** 10.3390/s26144424

**Published:** 2026-07-12

**Authors:** Kaichao Lei, Hongjia Xu, Xin Ye, Jingying Yue, Ye Jiang, Shuqi Li, Yachao Zhang

**Affiliations:** 1 School of Science, Changchun Institute of Technology, Changchun 130012, China; leikaichao@ccit.edu.cn (K.L.); yuejy444@163.com (J.Y.); 2 Changchun Institute of Optics, Fine Mechanics and Physics, Chinese Academy of Sciences, Changchun 130033, China; xuhongjia24@mails.ucas.ac.cn (H.X.); yexin@ciomp.ac.cn (X.Y.); jiangye@ciomp.ac.cn (Y.J.); lishuqi@ciomp.ac.cn (S.L.); 3 School of Optoelectronics, University of Chinese Academy of Sciences, Beijing 100049, China

**Keywords:** monochromator, bandwidth, standard lamp-plaque

## Abstract

High-precision absolute radiometric calibration is the cornerstone of space-based quantitative remote sensing. Monochromator-based radiance calibration is a key approach for space-borne radiometric benchmark transfer; however, the influence of the monochromator bandwidth on calibration accuracy urgently requires validation via an independent, SI-traceable benchmark. Taking a spectrometer as a case study, this paper establishes an equivalent theoretical calibration model. The model reveals that discrepancies in the equivalent spectral distributions of the calibration sources primarily contribute to their calibration deviations. Within the 775–855 nm range, cross-comparison experiments were conducted between the monochromator facility and a reference standard lamp-plaque. Experimental results demonstrate that under bandwidth configurations of 2 nm, 4 nm, and 6 nm, relative deviations in calibrated radiance responsivity stabilize at approximately 2.3%, exhibiting no significant dependence on bandwidth variations. Crucially, the consistency between the two methodologies is rigorously verified, as the radiance responsivity ratios uniformly fall within the combined expanded measurement uncertainty bounds. By demonstrating that bandwidth expansion within the specified 2–6 nm range does not degrade calibration accuracy, this study provides a critical empirical basis for safely increasing solar monochromator bandwidths to improve the signal-to-noise ratio (SNR) in next-generation benchmark missions like the Space-based Radiometric Measurement Benchmark Mission (LIBRA) program.

## 1. Introduction

In recent years, space-based Earth observation technology has been undergoing a profound transformation from qualitative imaging to high-precision quantitative remote sensing [1,2]. High-precision quantitative remote sensing observation data serve as the core foundation for accurately assessing global climate change and meticulously parsing the physical mechanisms of the Earth’s radiation budget [3,4]. Within the full-link data processing framework of quantitative remote sensing, absolute radiometric calibration represents a critical link for reliably converting the digital numbers (DN) acquired by optical remote sensing payloads into absolute spectral radiance or reflectance [5,6]. However, with the increasingly stringent requirements imposed by modern remote sensing climate science applications on the absolute stability and measurement accuracy of observation data, traditional traceability frameworks-which rely on pre-launch laboratory calibration and on-orbit vicarious calibration-struggle to satisfy the escalating demands for low radiometric measurement uncertainty [7].

To significantly improve the measurement accuracy of on-orbit remote sensing instruments, the international community has successively initiated strategic aerospace missions aimed at establishing “space-based absolute radiometric measurement benchmarks.” Prominent examples of these initiatives include the Calibration Absolute Radiance and Refractivity Observatory (CLARREO) program of the United States [8,9] and the LIBRA mission steadily advancing in China [10]. Additionally, the Traceable Radiometry Underpinning Terrestrial- and Helio-Studies (TRUTHS) mission was previously proposed by the United Kingdom, though its development was cancelled at the end of 2025 [11,12]. The overarching vision of these space programs is to deploy “calibration satellites” equipped with instruments of extremely high measurement accuracy, thereby positioning measurement benchmarks with direct International System of Units (SI) traceability and absolute radiometric uncertainties of better than 1% into space orbits. This architecture ultimately enables high-frequency, ultra-high-precision on-orbit cross-calibration and value transfer for other Earth observation satellites [13].

To achieve the stringent <1% absolute radiometric calibration uncertainty required by these benchmark missions, the selection and characterization of the calibration source are critical. Currently, standard lamp-plaques and tunable lasers are two prominent calibration sources, yet both face practical limitations in continuous full-spectrum space-borne applications. Standard lamp-plaques provide a continuous broadband spectrum and direct SI traceability; however, limited by their spectral flux—particularly the weak optical radiation flux in the ultraviolet to blue bands—the signal-to-noise ratio (SNR) and accuracy during calibration are inherently restricted [14]. Alternatively, tunable lasers offer extremely high wavelength accuracy and high power. Despite these advantages, a single tunable laser covers a limited spectral range. Furthermore, their complex debugging processes, strict operating environment requirements, and inherent interference speckle effects pose significant challenges for operational, continuous full-spectrum remote sensor calibration [15,16].

In contrast, monochromator-based radiance calibration systems effectively overcome the aforementioned limitations. By utilizing a grating to disperse broadband sources, monochromators offer the unique advantages of continuous wavelength tunability across a wide spectral range, high dispersion, and adjustable spectral resolution [17,18]. The solar monochromator has been established as the core dispersive device for space-borne radiometric benchmark transfer in missions like the Earth–Moon Imaging Spectrometer of the LIBRA project.

In the calibration chain of the LIBRA mission, the signal registered by the spectrometer under calibration is inherently the convolution of the spectral line shape function output by the monochromator and the spectrometer’s own Instrumental Spectral Response Function (ISRF) [19]. The spectral line shape output by the monochromator is non-ideal; influenced by its internal slit function and the spectral distribution of the incident light source, variations in the monochromator’s intrinsic parameters directly affect the radiometric calibration results of the spectrometer [20]. Although recent studies have addressed the influence of parameters like monochromator bandwidth on calibration accuracy, these investigations have predominantly focused on the sensitivity analysis of internal parameters within the monochromator system itself [21].

In practical space-borne engineering, a critical trade-off exists between the monochromator bandwidth and the instrument’s SNR. While expanding the bandwidth significantly enhances the output energy and the corresponding SNR of the spectrometer, its potential degradation effect on absolute calibration accuracy remains a major concern. Consequently, there is an urgent lack of experimental evidence using an independent, SI-traceable external benchmark to directly validate whether the calibration accuracy remains consistent under these varying bandwidth configurations.

To address this gap and clearly establish the rigorous theoretical rationale for bandwidth optimization, this paper proposes a cross-verification method based on an SI-traceable standard lamp-plaque system, taking an SVC spectrometer as a case study. Calibrated by the National Institute of Metrology (NIM) of China, the spectral radiance of the standard lamp-plaque system is directly traceable to the national primary radiometric benchmark, thereby serving as an independent external reference. First, an equivalent calibration model for the monochromator-based spectral radiance calibration system is established to elucidate the physical origin of the discrepancies between monochromator- and lamp-plaque-based calibrations. Subsequently, a high-precision monochromator spectral radiance calibration system is constructed. Using the standard lamp-plaque system as the baseline reference, cross-comparison experiments are conducted under spectral bandwidths of 2 nm, 4 nm, and 6 nm. The consistency between the two calibration methodologies is rigorously verified, as the radiance responsivity ratios uniformly fall within the combined expanded measurement uncertainty bounds. This unequivocally proves that variations in bandwidth exert no significant influence on the calibration consistency. This study provides crucial engineering guidance for the absolute calibration scheme design of spectrometers in space-based radiometric benchmark missions such as the LIBRA project.

## 2. Theoretical Analysis

### 2.1. Calibration Principle of the Monochromator-Based Spectral Radiance Calibration System

In a conventional monochromator-based spectral radiance calibration system, the integrated radiance responsivity is traditionally acquired through discrete wavelength scanning. However, to directly cross-verify this dynamic, narrow-band scanning process against a static, continuous broad-spectrum source (such as an SI-traceable standard lamp-plaque), an equivalent theoretical bridge must be constructed.

Assuming the spectral bandwidth set by the monochromator is Δλ and the center wavelength is $\lambda^{\prime }$, the spectral radiance of its output quasi-monochromatic light is denoted as $L_{\Delta \lambda }(\lambda^{\prime },\;\lambda )$. The absolute instrumental spectral response function of the spectrometer channel $\lambda_{0}$ is characterized as $R(\lambda_{0},\;\lambda )$. Accordingly, the response of the spectrometer channel $\lambda_{0}$ to the quasi-monochromatic light centered at $\lambda^{\prime }$ output by the monochromator is:

(1) $${\mathrm{DN}}_{\mathrm{mono}}\left(\lambda_{0}\;,\;\lambda^{\prime }\right)=\int_{\lambda_{\min}}^{\lambda_{\max}}R(\lambda_{0},\;\lambda )L_{\Delta \lambda }(\lambda^{\prime },\;\lambda )d\lambda$$

where $\left[\lambda_{\min},\lambda_{\max}\right]=\left[\lambda_{\min}^{\mathrm{mono}},\lambda_{\max}^{\mathrm{mono}}\right]\cap \left[\lambda_{\min}^{\mathrm{spec}},\lambda_{\max}^{\mathrm{spec}}\right]$; $\left[\lambda_{\min}^{\mathrm{mono}},\lambda_{\max}^{\mathrm{mono}}\right]$ denotes the wavelength range of $L_{\Delta \lambda }(\lambda^{\prime },\;\lambda )$ and $\left[\lambda_{\min}^{\mathrm{spec}},\lambda_{\max}^{\mathrm{spec}}\right]$ denotes that of $R(\lambda_{0},\;\lambda )$.

Instead of treating the aforementioned calibration process as a routine summation of independent monochromatic responses, our proposed model mathematically transforms this temporal scanning into a virtual equivalent spatial source. The core of this construction lies in the precise modeling of the convolution mechanism. In our model, the underlying slit function is not assumed to be a fixed flat-top distribution; rather, it represents dynamic changes based strictly on the specific monochromator line shape.

By keeping the monochromator bandwidth fixed and continuously tuning its central wavelength, the integrated radiance responsivity of the spectrometer channel calibrated by the monochromator is obtained by integrating the response over the complete tuning range:

(2) $$R_{\Delta \lambda }(\lambda_{0})\;=\;\int_{\lambda_{\min}^{\prime }}^{\lambda_{\max}^{\prime }}\frac{{\mathrm{DN}}_{\mathrm{mono}}\left(\lambda_{0}\;,\;\lambda^{\prime }\right)}{\int_{\lambda_{\min}}^{\lambda_{\max}}L_{\Delta \lambda }(\lambda^{\prime },\;\lambda )d\lambda }d\lambda^{\prime }=\int_{\lambda_{\min}^{\prime }}^{\lambda_{\max}^{\prime }}\frac{\int_{\lambda_{\min}}^{\lambda_{\max}}R(\lambda_{0},\;\lambda )L_{\Delta \lambda }(\lambda^{\prime },\;\lambda )d\lambda \;}{\int_{\lambda_{\min}}^{\lambda_{\max}}L_{\Delta \lambda }(\lambda^{\prime },\;\lambda )d\lambda }d\lambda^{\prime }$$

where $\left[\lambda_{\min}^{\prime },\lambda_{\max}^{\prime }\right]$ represents the tuning range of the monochromator’s central wavelength.

By interchanging the order of integration, this calibration paradigm is uniquely parameterized by a synthesized weight function, which we define as the equivalent synthesized spectral distribution, $L_{\sum }\left(\lambda \right)$:

(3) $$L_{\sum }(\lambda )=\int_{\lambda_{\min}^{\prime }}^{\lambda_{\max}^{\prime }}\frac{L_{\Delta \lambda }(\lambda^{\prime },\;\lambda )}{\int_{\lambda_{\min}}^{\lambda_{\max}}L_{\Delta \lambda }(\lambda^{\prime },\;\lambda )d\lambda }\;d\lambda^{\prime }$$

It is crucial to highlight the unique theoretical design of this constructed parameter. Within our theoretical framework, $L_{\sum }\left(\lambda \right)$ cannot be treated as mathematically equivalent to a generic spectral distribution ratio. Because it intrinsically couples the dynamic slit function and the instrument’s spectral response across the integration domain, rigorous analytical derivation dictates that the integral value of $L_{\sum }\left(\lambda \right)$ over the full spectral band is not 1.

Using this carefully constructed equivalent spectrum, the final radiance responsivity calibration via a monochromator can be concisely mapped to the broadband calibration equation:

(4) $${\;R}_{\Delta \lambda }(\lambda_{0})=\;\int_{\lambda_{\min}}^{\lambda_{\max}}R(\lambda_{0},\;\lambda )\cdot \;L_{\sum }(\lambda )d\lambda \;$$

This modeling framework offers a demonstrable theoretical innovation: it physically proves that the discrete monochromator scanning process is mathematically equivalent to a direct calibration utilizing a virtual broadband source entirely governed by $L_{\sum }\left(\lambda \right)$, thereby laying a rigorous theoretical foundation for independent benchmark cross-verification.

### 2.2. Calibration Principle Based on the SI-Traceable Standard Lamp-Plaque System

To rigorously validate the equivalent theoretical model proposed in Section 2.1, an SI-traceable standard lamp-plaque system is introduced as the absolute baseline reference.

Unlike the monochromator-based system, the standard lamp-plaque acts as a static, broadband Lambertian source. Assuming the spectral radiance of the standard lamp-plaque is $L_{\mathrm{lamp}}\left(\lambda \right)$, the absolute radiance responsivity of the spectrometer channel $\lambda_{0}$ calibrated by this physical source can be directly derived as:

(5) $${\;R}_{\mathrm{lamp}}(\lambda_{0})=\;\int_{\lambda_{\min}^{\mathrm{spec}}}^{\lambda_{\max}^{\mathrm{spec}}}R(\lambda_{0},\;\lambda )\frac{L_{\mathrm{lamp}}\left(\lambda \right)}{L_{\mathrm{lamp}}(\lambda_{0})}d\lambda$$

Comparing Equation (5) with the monochromator equivalent model (Equation (4)) reveals a fundamental discrepancy in their underlying mathematical formulations and physical mechanisms. Specifically, the equivalent synthesized spectral distribution $L_{\sum }\left(\lambda \right)$ in the monochromator system is a mathematically constructed, dimensionless weight function derived from the dynamic scanning of discrete slit functions. In contrast, the lamp-plaque calibration intrinsically relies on the relative spectral radiance distribution, $\frac{L_{\mathrm{lamp}}\left(\lambda \right)}{L_{\mathrm{lamp}}(\lambda_{0})}$, which represents the continuous physical power spectrum of an authentic broadband source. Because of this inherent physical authenticity and its direct SI traceability, the lamp-plaque system serves as an ideal independent benchmark to verify whether the virtual equivalent spectrum $L_{\sum }\left(\lambda \right)$ introduces any theoretical or practical calibration distortions.

### 2.3. Numerical Simulation and Assessment of Spectral Mismatch

To verify the discrepancy in the radiance responsivity of the spectrometer calibrated by the lamp-plaque and the monochromator under different bandwidths, this section takes the SVC spectrometer as an example and utilizes the 833.5 nm channel for theoretical calculation to analyze the difference between the two calibration methods.

In this study, the radiance responsivity function of the 833.5 nm channel of the SVC spectrometer was calibrated using a tunable laser-based radiance calibration system, covering a wavelength range of 750 nm to 940 nm with a laser wavelength tuning step set to 0.5 nm. The spectral bandwidth of the 833.5 nm channel of the SVC spectrometer is approximately 4 nm. The wavelength tuning step adopted in this calibration is only 1/8 of this spectral bandwidth, allowing for the precise acquisition of the radiance responsivity function R(833.5, λ) for the 833.5 nm channel. Since the morphological shape of the monochromator slit function has a minimal impact on the radiance responsivity calibration results of the SVC spectrometer [22], this paper employs a normalized Gaussian function to replace the monochromator slit function, as shown in Equation (6):

(6) $${\;L}_{\Delta \lambda }(\lambda^{\prime },\;\lambda )=\frac{1}{\sqrt{2\pi }\sigma }e^{-\;\frac{{\left(\lambda -\lambda^{\prime }\right)}^{2}}{2\sigma^{2}}}=\frac{1}{\sqrt{2\pi }(\Delta \lambda /2\sqrt{2\ln2})}e^{-\;\frac{{\left(\lambda -\lambda^{\prime }\right)}^{2}}{2{(\Delta \lambda /2\sqrt{2\ln2})}^{2}}}$$

where $L_{\Delta \lambda }(\lambda^{\prime },\;\lambda )$ represents the quasi-monochromatic light output by the monochromator, $\lambda^{\prime }$ is the central wavelength of the monochromator, Δλ is the bandwidth of the quasi-monochromatic light output by the monochromator, and $\sigma =\Delta \lambda /2\sqrt{2\ln2}$ is the standard deviation of the normalized Gaussian function.

The spectral radiance values of the standard lamp-plaque used for calibration are calibrated and traceable, covering a spectral range of 250 nm to 2500 nm with a nominal wavelength interval of 10 nm. Finally, a linear interpolation method was used to uniformly match the wavelength coordinates among the tunable laser, the SVC spectrometer, and the standard lamp-plaque, thereby completing the wavelength benchmark alignment.

Substituting three sets of quantities—the spectral radiance responsivity of the SVC spectrometer’s 833.5 nm channel, the normalized Gaussian function that simulates monochromator output under various bandwidths, and the relative spectral radiance of the standard lamp plaque into Equation (2) and Equation (5), we derive the channel’s radiance responsivity calibrated by the monochromator and lamp plaque, as shown in Equations (7) and (8):

(7) $${\;R}_{\Delta \lambda }(833.5)\;=\int_{\lambda_{\min}}^{\lambda_{\max}}R(833.5,\;\lambda )\left[\int_{\lambda_{\min}^{\prime }}^{\lambda_{\max}^{\prime }}\frac{\frac{1}{\sqrt{2\pi }\sigma }e^{-\;\frac{{\left(\lambda -\lambda^{\prime }\right)}^{2}}{2\sigma^{2}}}}{\int_{\lambda_{\min}}^{\lambda_{\max}}\frac{1}{\sqrt{2\pi }\sigma }e^{-\;\frac{{\left(\lambda -\lambda^{\prime }\right)}^{2}}{2\sigma^{2}}}d\lambda }\;d\lambda^{\prime }\right]d\lambda \;$$

(8) $${\;R}_{\mathrm{lamp}}(833.5)=\int_{\lambda_{\min}^{\mathrm{spec}}}^{\lambda_{\min}^{\mathrm{spec}}}R(833.5,\;\lambda )\frac{L_{\mathrm{lamp}}\left(\lambda \right)}{L_{\mathrm{lamp}}(\lambda_{0})}d\lambda \;$$

Since it is difficult to fit the radiance responsivity data of the SVC spectrometer’s 833.5 nm channel into an exact function, the integral operations in Equations (7) and (8) are converted into numerical integration operations. Equations (9) and (10) are used to calculate the radiance responsivity of the 833.5 nm channel calibrated by the monochromator and the lamp-plaque, respectively.

(9) $${\;R}_{\Delta \lambda }(833.5)=\sum_{\lambda^{\prime }=750}^{940}\sum_{\lambda =750}^{940}\frac{R(833.5,\;\lambda )\frac{1}{\sqrt{2\pi }\sigma }e^{-\;\frac{{\left(\lambda -\lambda^{\prime }\right)}^{2}}{2\sigma^{2}}}}{\int_{\lambda_{\min}}^{\lambda_{\max}}\frac{1}{\sqrt{2\pi }\sigma }e^{-\;\frac{{\left(\lambda -\lambda^{\prime }\right)}^{2}}{2\sigma^{2}}}d\lambda }$$

(10) $${\;R}_{\mathrm{lamp}}(833.5)=\sum_{\lambda =750}^{940}\frac{R(833.5,\;\lambda )\;L_{\mathrm{lamp}}\left(\lambda \right)}{L_{\mathrm{lamp}}(\lambda_{0})}$$

The bandwidth Δλ of the quasi-monochromatic light output by the monochromator is set to 2 nm, 4 nm, and 6 nm, respectively, while the wavelength scanning interval is fixed at 0.5 nm. By substituting these into Equation (9), the radiance responsivity of the SVC spectrometer’s 833.5 nm channel calibrated by the monochromator under different bandwidths is calculated. Taking the radiance responsivity calibrated by the standard lamp-plaque calculated using Equation (10) as the reference standard, the relative deviations of the radiance responsivities calibrated by the monochromator at bandwidths of 2 nm, 4 nm, and 6 nm are compared against it, as shown in Table 1.

Table 1 indicates that there is a relative deviation of approximately 0.31% between the radiance responsivity of the 833.5 nm channel calibrated by the monochromator under different bandwidths and that calibrated by the lamp-plaque. The calculation results demonstrate that the primary cause of the discrepancy in the radiance responsivity calibrated by the monochromator and the standard lamp-plaque is the difference in the equivalent spectral distributions of the two light sources, whereas the influence of the monochromator bandwidth is minimal.

## 3. Experimental Validation

Taking the SVC spectrometer as an example, this paper utilizes the monochromator-based spectral radiance calibration system and the standard lamp-plaque system, respectively, to calibrate the radiance responsivity of the SVC spectrometer, thereby verifying whether the radiance responsivities calibrated by the two methodologies exhibit consistency.

### 3.1. Monochromator-Based Spectral Radiance Calibration System

**Figure 1:**
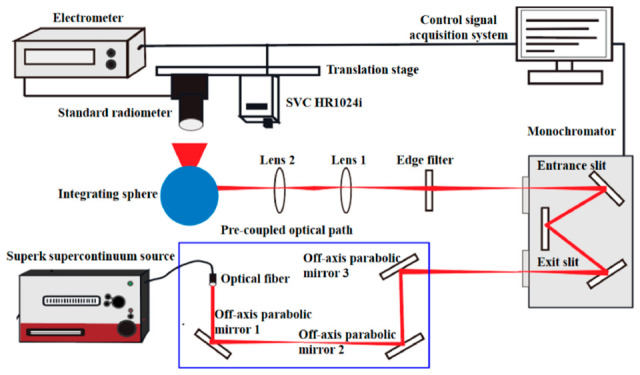
Schematic diagram of the monochromator-based spectral radiance calibration system.

The monochromator-based spectral radiance calibration system is illustrated in Figure 1. A SuperK EXB-6 supercontinuum (NKT Photonics A/S, Birkerød, Denmark) source serves as the system source, covering a wavelength range of 400 nm to 2000 nm. The front-end coupling optical path consists of three off-axis parabolic (OAP) mirrors with focal lengths of 25.4 mm, 101.6 mm, and 50.8 mm, respectively, enabling high-efficiency coupling between the supercontinuum source and the monochromator. The output beam diameter of the supercontinuum source is approximately 2 mm; after beam shaping by the coupling optics, the spot diameter at a position 25.4 mm away from the center of the third OAP mirror is better than 1 mm. The F-number of the front-end coupling optics is approximately 6.2, while that of the monochromator is about 4.2. Consequently, the output light from the coupling optics can be completely received by the monochromator, minimizing energy loss. The monochromator model is Omni-λ3009i (Zolix Instruments Co., Ltd., Beijing, China), which contains three built-in gratings with groove densities of 1200 lines/mm @ 500 nm, 600 lines/mm @ 1000 nm, and 600 lines/mm @ 1600 nm, covering a wavelength range from roughly 330 nm to 3000 nm. Both the entrance and exit slit widths of the monochromator are adjustable; by regulating these widths, the monochromator can achieve a quasi-monochromatic light output with a bandwidth ranging from 2 nm to 6 nm. An edge filter with an optical density (OD) greater than 3 and an edge wavelength of 650 nm is used to suppress the influence of second-order spectra. Two lenses with focal lengths of 50 mm and 200 mm, respectively, are employed to shape the quasi-monochromatic light emerging from the monochromator, ensuring that the beam diameter at the entrance of the integrating sphere is smaller than the entrance aperture diameter, so that all output light from the monochromator is collected by the integrating sphere. The integrating sphere has a diameter of 150 mm and is constructed with PTFE inner wall material, which exhibits a reflectivity greater than 98% in the 700 nm to 900 nm wavelength range to minimize energy loss. A standard radiometer serves as the radiance benchmark for the system; its radiance responsivity is traceable to the NIM, and its measurement range covers wavelengths from 700 nm to 900 nm.

### 3.2. Experimental Protocol of the Monochromator-Based Spectral Radiance Calibration System

First, the supercontinuum source is turned on and allowed to warm up for approximately 1 h. The laser output from the supercontinuum source is collected by the monochromator through the coupling optical path. The entrance and exit slit widths of the monochromator are adjusted to 1 mm, and a grating with a groove density of 1200 lines/mm @ 500 nm is selected; at this configuration, the output quasi-monochromatic light from the monochromator has a bandwidth of approximately 2 nm. The central wavelength of the quasi-monochromatic light emitted by the monochromator is tuned to $\lambda^{\prime }$, and the radiation is directed into the integrating sphere through two lenses. The standard radiometer and the SVC spectrometer are mounted on a translation stage, positioned approximately 10 mm away from the exit port of the integrating sphere, and are configured to be co-axial and at the same height as the exit port. The standard radiometer first measures the spectral radiance at the integrating sphere exit port. Assuming that the current output from the standard radiometer measured by the electrometer is $I_{R}(\lambda^{\prime })$ and the radiance responsivity of the standard radiometer at wavelength $\lambda^{\prime }$ is $R_{R}(\lambda^{\prime })$, the spectral radiance at the integrating sphere exit port measured by the standard radiometer is expressed as:

(11) $${\;L}_{\mathrm{sphere}}(\lambda^{\prime })=I_{R}(\lambda^{\prime })/R_{R}(\lambda^{\prime })$$

Subsequently, the standard radiometer is translated away, and the SVC spectrometer measures the spectral radiance at the integrating sphere exit port. Assuming that the integration-time-normalized DN output from the i-th channel of the SVC spectrometer is ${\mathrm{DN}}_{i}\left(\lambda \right)$, the radiance responsivity of the i-th channel of the SVC spectrometer calibrated by the quasi-monochromatic light centered at $\lambda^{\prime }$ can be formulated as:

(12) $${\;R}_{i}^{\mathrm{mono}}(\lambda^{\prime })={\mathrm{DN}}_{i}(\lambda^{\prime })/L_{\mathrm{sphere}}(\lambda^{\prime })\;$$

Next, the central wavelength of the quasi-monochromatic light output by the monochromator is continuously adjusted within the wavelength range of 765 nm to 865 nm, with a tuning interval of approximately 0.5 nm. Since the spectral bandwidth of the SVC spectrometer channels within this spectral range is approximately 4 nm, the wavelength tuning step size adopted in this calibration is only 1/8 of the instrumental bandwidth, which enables the precise profiling of the radiance responsivity function of the SVC spectrometer channels. By repeating the aforementioned operations, the monochromator-calibrated radiance responsivity of the i-th channel of the SVC spectrometer within the 765 nm to 865 nm wavelength range can be obtained:

(13) $${\;R}_{i}^{\mathrm{mono}}=\sum_{\lambda_{min}^{\prime }}^{\lambda_{max}^{\prime }}R_{\mathrm{SVC}\;i}(\lambda^{\prime })\cdot \Delta \lambda$$

where $\lambda_{min}^{\prime }$ and $\lambda_{max}^{\prime }$ represent the lower and upper limits of the monochromator’s wavelength tuning range, respectively, and Δλ denotes the wavelength tuning interval of the monochromator.

**Figure 2:**
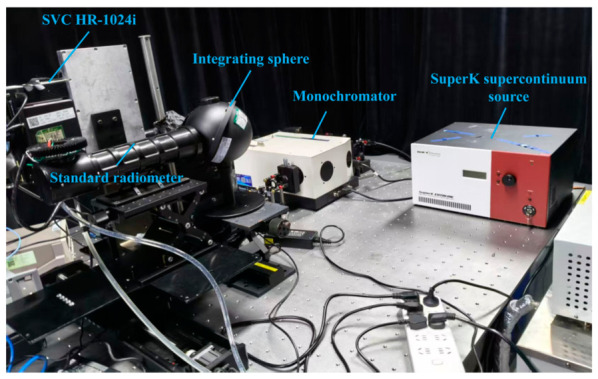
Experimental setup of the monochromator-based spectral radiance calibration system.

Finally, with the selected grating of the monochromator held constant, the widths of the entrance and exit slits are sequentially adjusted to 2 mm and 3 mm to generate quasi-monochromatic light with bandwidths of approximately 4 nm and 6 nm, respectively. By repeating the three operational steps described above, the radiance responsivities of the SVC spectrometer calibrated by the monochromator-emitted quasi-monochromatic light at bandwidth configurations of 2 nm, 4 nm, and 6 nm are determined. The experimental setup is shown in Figure 2.

### 3.3. Calibration of SVC Spectrometer Radiance Responsivity Using a Standard Lamp-Plaque System

**Figure 3:**
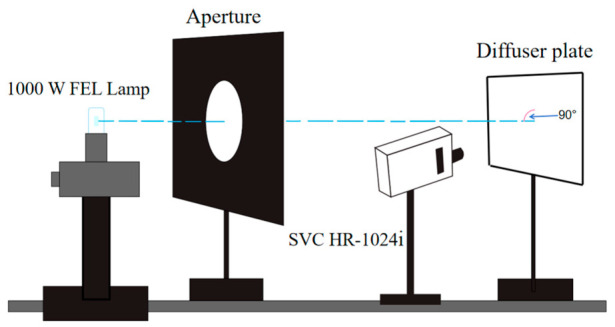
Schematic diagram of the standard lamp-plaque spectral radiance calibration system.

The standard lamp-plaque spectral radiance calibration system primarily consists of a 1000 W tungsten halogen lamp, a diffuse reflectance plaque, and a stray light baffle, as schematically illustrated in Figure 3. The stray light baffle are utilized to mitigate the impact of stray light on the calibration results. The optical axis of the 1000 W tungsten halogen lamp coincides with the normal of the diffuse reflectance plaque, and the filament center is at the same height as the center of the plaque, allowing the emitted light to normally illuminate the plaque surface. Upon reflection by the diffuse plaque, a uniform Lambertian surface source is formed, providing an absolute spectral radiance benchmark for the SVC spectrometer. The diffuse plaque is positioned at a distance of 50 cm from the tungsten halogen lamp. This distance is identical to the standard distance employed by the NIM during lamp spectral irradiance calibration, thereby avoiding additional errors introduced by calculating the lamp irradiance via the inverse-square law. Assuming that the spectral irradiance calibrated by NIM at the standard distance is E(λ), the bidirectional reflectance factor of the diffuse plaque is $R(0^{{}^{\circ}}/45^{{}^{\circ}},\lambda )$, and the measured distance between the tungsten halogen lamp and the diffuse plaque is r, the spectral radiance of the standard lamp-plaque system can be expressed as:

(14) $${\;L}_{\mathrm{lamp}}(\lambda )=\frac{R(0^{{}^{\circ}}/45^{{}^{\circ}},\;\lambda )}{\pi }\cdot {\left(\frac{r}{r_{o}}\right)}^{2}\cdot E_{\mathrm{lamp}}(\lambda )\;$$

**Figure 4:**
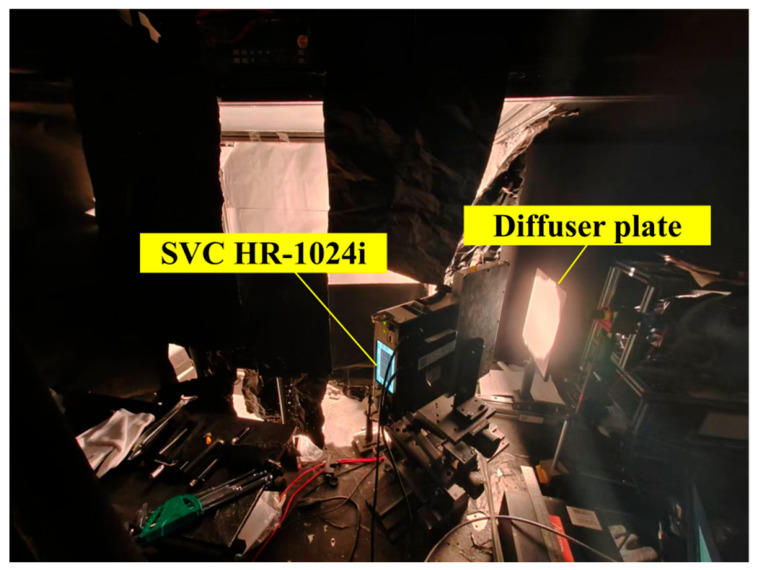
Experimental layout for the radiance responsivity calibration of the SVC spectrometer based on the standard lamp-plaque configuration.

For the calibration run, the SVC spectrometer is leveled with the center of the diffuse plaque, its optical axis anchored on the plaque center at a 45° observation angle relative to the plaque normal-a configuration that flawlessly replicates the baseline alignment geometry of the bidirectional reflectance factor. The physical separation between the spectrometer and the plaque surface is maintained at approximately 250 mm, as schematically detailed in Figure 4. The baseline radiance dataset for the reference standard lamp-plaque assembly is certified by NIM across a broad spectral window from 250 nm to 2500 nm at discrete 10 nm increments. Centering our analysis on the 775 nm to 855 nm spectral band of the SVC spectrometer, a linear interpolation routine is executed to map the exact reference radiance values onto the specific central wavelengths of the spectrometer’s individual channels. Given that the interpolated reference radiance at the central wavelength of the i-th channel is $L_{\mathrm{lamp}}(\lambda_{i})$ and the corresponding integration-time-normalized DN captured by the SVC instrument is ${\mathrm{DN}}_{i}\left(\lambda \right)$, the absolute radiance responsivity of the spectrometer’s i-th channel under the lamp-plaque paradigm is formulated as:

(15) $${\;R}_{i}^{\mathrm{lamp}}=\frac{{\mathrm{DN}}_{i}(\lambda )\;}{L_{\mathrm{lamp}}(\lambda_{i})}$$

## 4. Experimental Results and Analysis

### 4.1. Experimental Results

**Figure 5:**
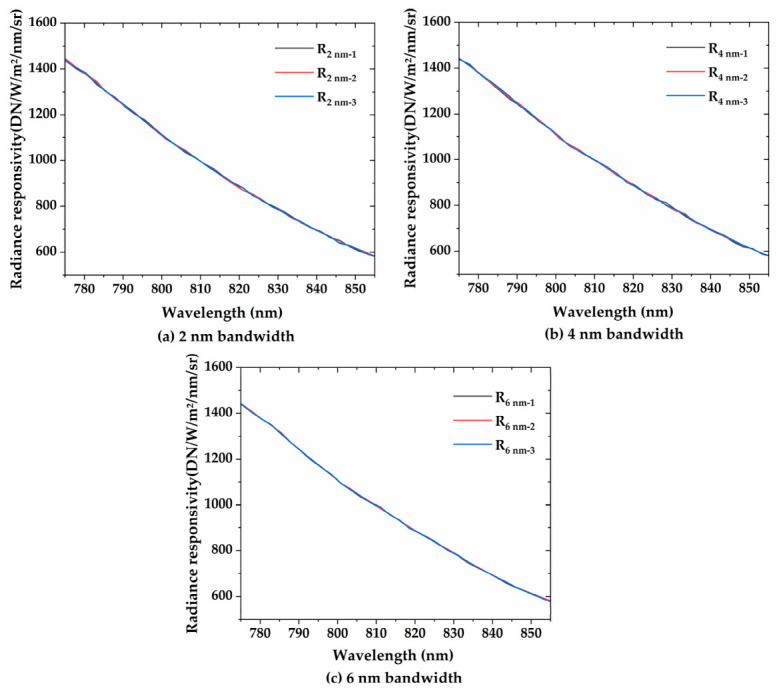
Radiance responsivity curves of the SVC spectrometer calibrated by the monochromator system under 2 nm, 4 nm, and 6 nm bandwidth configurations.

In the monochromator-based radiance calibration experiment, the central wavelength output by the monochromator is scanned and regulated within the range of 765 nm to 865 nm, and the single-channel spectral bandwidth of the SVC spectrometer is approximately 4 nm. Referring to Equation (11), the radiance responsivity of the i-th channel of the SVC spectrometer is the integrated result of the calibration contributions from the incident monochromatic light at each wavelength within the spectral response window of that channel. Restricted by the energy distribution of the monochromator output, the signal-to-noise ratio (SNR) of the spectrometer’s output light signal is insufficient at the peripheral bands of the scanning wavelength, failing to satisfy the requirements for calibration accuracy. Consequently, the calibration data for these spectral channels are discarded in subsequent data processing. Figure 5 illustrates the experimental results of the SVC spectrometer’s radiance responsivity calibrated by the monochromator within the 775 nm to 855 nm wavelength range.

**Figure 6:**
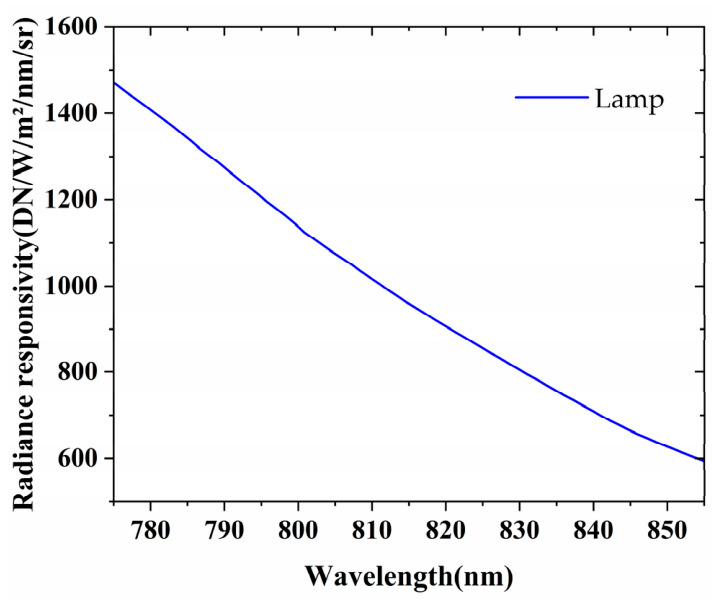
Radiance responsivity curves of the SVC spectrometer calibrated by the standard lamp-plaque system.

Distinct from the wavelength-by-wavelength scanning calibration principle of the monochromator, the standard lamp-plaque system serves as a broadband light source whose output spectral radiance spans from 250 nm to 2500 nm, fully covering all spectral channels of the SVC spectrometer in the visible wavelength band. This calibration methodology enables the simultaneous absolute calibration of all spectral channels without the need for wavelength-by-wavelength scanning or integral conversion across the channel response intervals. Because its spectral radiance values are directly traceable to the national primary radiometric benchmark of the NIM, the radiance responsivity of each channel of the SVC spectrometer determined via the standard lamp-plaque calibration represents the absolute responsivity of the instrument. The radiance responsivity results of the SVC spectrometer channels within the 775 nm to 855 nm wavelength range calibrated by the standard lamp-plaque system are depicted in Figure 6.

**Figure 7:**
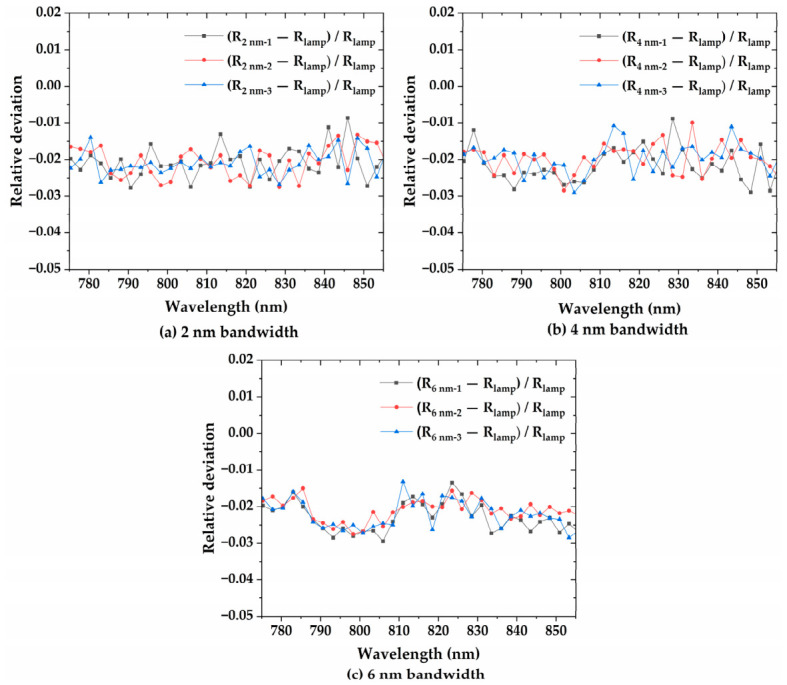
Relative deviation of the radiance responsivity calibrated by the monochromator system compared against the standard lamp-plaque baseline under 2 nm, 4 nm, and 6 nm bandwidth configurations.

Taking the radiance responsivity calibrated by the standard lamp-plaque system as the reference standard, the relative deviation between the radiance responsivities calibrated by the monochromator and the standard lamp-plaque system is calculated according to Equation (16), as displayed in Figure 7.

(16) $$E=\frac{{{\;R}_{i}^{\mathrm{mono}}-R}_{i}^{\mathrm{lamp}}}{{\;R}_{i}^{\mathrm{lamp}}}$$

The experimental results demonstrate that under different bandwidth configurations, the relative deviation between the monochromator radiance calibration system and the standard lamp-plaque system in calibrating the SVC spectrometer’s radiance responsivity stabilizes at approximately 2.3%. In this study, three independent and complete calibration runs were performed under each bandwidth configuration of 2 nm, 4 nm, and 6 nm. The relative deviations of the radiance responsivity measured across all channels of the SVC spectrometer were better than 0.9%, demonstrating excellent measurement repeatability and stability of the system. Furthermore, a one-way analysis of variance (ANOVA) was conducted on the calibration deviations across the three bandwidth conditions. The statistical results reveal that the *p*-values for all spectral channels are consistently greater than 0.1, remaining well above the 0.05 significance threshold. Given the sample size and significance level of this study, no statistically significant difference was detected among the 2 nm to 6 nm bandwidth configurations. These results conclusively demonstrate that the absolute calibration accuracy in this experiment has no significant correlation with bandwidth variations.

### 4.2. Uncertainty Analysis

The standard lamp-plaque system is provided by the NIM, and its uncertainty is evaluated by the NIM. Within the wavelength range of 700 nm to 900 nm, the uncertainty of the standard lamp-plaque is approximately 2.5% (k = 1). In the calibration experiment of the SVC spectrometer’s radiance responsivity using the standard lamp-plaque system, the measurement repeatability of the SVC spectrometer is better than 0.5% (k = 1). Therefore, the combined uncertainty of the radiance responsivity calibration via the standard lamp-plaque system is approximately 2.55% (k = 1).

The following sections focus on analyzing the measurement uncertainty of the SVC spectrometer’s radiance responsivity calibrated by the monochromator-based spectral radiance calibration system.

#### 4.2.1. Measurement Repeatability of the Standard Radiometer

The stability of the supercontinuum source is the governing factor affecting the measurement repeatability of the standard radiometer. Utilizing the supercontinuum source as the experimental illumination, the standard radiometer continuously measures for approximately 30 min, yielding a measurement repeatability of better than 0.4% (k = 1).

#### 4.2.2. Radiance Responsivity of the Standard Radiometer

The uncertainty of the radiance responsivity of the NIM reference radiometer and the uncertainty of the standard radiometer when measuring quasi-monochromatic light are the primary sources contributing to the radiance responsivity uncertainty of the standard radiometer.

(a) The NIM reference radiometer: The standard radiometer in the monochromator calibration system is calibrated by the NIM reference radiometer. The source used for calibration is a Spectra-Physics tunable laser. During the calibration process, the measurement repeatability of the standard radiometer is less than 0.1% (k = 1), and the uncertainty of the NIM reference radiometer is approximately 0.3% (k = 1). Consequently, the uncertainty of the standard radiometer’s radiance responsivity is determined to be approximately 0.32% (k = 1).

(b) Quasi-monochromatic light: The source for the radiance responsivity calibration experiment of the standard radiometer is a Spectra-Physics laser with a bandwidth on the picometer (pm) scale. In contrast, the source of the monochromator radiance calibration system is the quasi-monochromatic light produced by the supercontinuum source passing through the monochromator, which features a bandwidth on the nanometer (nm) scale. Since the latter is significantly broader than the laser bandwidth, it is necessary to analyze the discrepancy in the radiance responsivity of the standard radiometer when measuring these two types of sources.

The radiance responsivity of the standard radiometer when measuring quasi-monochromatic light can be expressed as follows:

(17) $${\;R}_{\Delta \lambda }(\lambda )\;=\;\frac{\int_{\lambda_{\min}}^{\lambda_{\max}}R_{\mathrm{SP}}\left(\lambda \right)L_{\Delta \lambda }(\lambda )d\lambda }{\int_{\lambda_{\min}}^{\lambda_{\max}}L_{\Delta \lambda }(\lambda )d\lambda }\;$$

where $R_{\mathrm{SP}}\left(\lambda \right)$ is the radiance responsivity of the standard radiometer when the source is a laser; $L_{\Delta \lambda }\left(\lambda \right)$ is the spectral radiance of the quasi-monochromatic light centered at λ; and $\left[\lambda_{\min},\lambda_{\max}\right]$ represents the intersection between the wavelength range of the quasi-monochromatic light output by the monochromator and the corresponding wavelength range of $R_{\mathrm{SP}}\left(\lambda \right)$.

The quasi-monochromatic light output by the monochromator is represented by a normalized Gaussian function that best approximates its spectral line shape. Given the calibrated radiance responsivities of the standard radiometer at discrete wavelengths of 740.695 nm, 760.371 nm, 780.566 nm, 800.294 nm, 820.431 nm, 840.440 nm, and 870.364 nm, the radiance responsivity of the standard radiometer at any arbitrary wavelength within the 741 nm to 870 nm range can be computed via linear interpolation. The radiance responsivity of the standard radiometer is then calculated using Equation (18).

(18) $${\;R}_{\Delta \lambda }(\lambda )=\frac{\sum_{\lambda_{\min}}^{\lambda_{\max}}R_{\mathrm{SP}}(\lambda )\cdot L_{\Delta \lambda }(\lambda )\Delta \lambda }{\sum_{\lambda_{\min}}^{\lambda_{\max}}L_{\Delta \lambda }(\lambda )\Delta \lambda }$$

Taking the quasi-monochromatic light with a monochromator central wavelength of 800 nm as an example, where $\lambda_{\min}=\;785\;\mathrm{nm}$ and $\lambda_{\max}=\;815\;\mathrm{nm}$, the wavelength tuning interval is set to 0.5 nm. With the bandwidth of the quasi-monochromatic light held constant, the radiance responsivity of the standard radiometer when measuring the quasi-monochromatic light centered at 800 nm can be determined from Equation (18). The radiance responsivity of the standard radiometer when measuring an 800 nm laser can be obtained via linear interpolation, as tabulated in Table 2.

As indicated in Table 2, the difference in the radiance responsivity of the standard radiometer between quasi-monochromatic light and laser measurements is minimal and negligible. In summary, the measurement uncertainty of the radiance responsivity for the standard radiometer is approximately 0.32%.

#### 4.2.3. Measurement Repeatability of the SVC Spectrometer

The stability of the supercontinuum source is the primary factor affecting the measurement repeatability of the SVC spectrometer. Upon tuning the quasi-monochromatic light output by the monochromator to a specific wavelength, the SVC spectrometer performs 5 consecutive measurements, yielding a measurement repeatability of better than 0.4% (k = 1).

#### 4.2.4. Numerical Integration

The numerical integration method is employed to compute the radiance responsivity of a specific channel of the SVC spectrometer. The measurement uncertainty introduced by this method is predominantly governed by the profile shape of the SVC spectrometer’s radiance responsivity function and the wavelength tuning interval. The spectral line shape of the SVC spectrometer’s radiance responsivity function approximates a Gaussian function, and the wavelength tuning interval in the monochromator-based calibration experiment is approximately 0.5 nm. The measurement uncertainty introduced by calculating the radiance responsivity through numerical integration is better than 0.5% (k = 1).

#### 4.2.5. Temperature

A water-cooling system controls the temperature of the SVC spectrometer. Throughout the entire experimental process, the temperature variation of the SVC spectrometer detector is better than 0.2 °C, and the impact of temperature on the SVC spectrometer is negligible. The detector of the standard radiometer is an S2281 Si photodiode. The laboratory temperature variation throughout the experiment is better than 1 °C, and the measurement uncertainty introduced by the temperature of the standard radiometer is better than 0.1% (k = 1).

#### 4.2.6. Detector Linearity

The standard radiometer used in this study has its radiance responsivity traceable to the reference radiometer of the NIM. The measurement signals of the standard radiometer differ by two orders of magnitude between its own radiance responsivity calibration experiment and the radiance responsivity calibration experiment of the SVC spectrometer. The detector of the standard radiometer is a Si photodiode with excellent linearity, and the measurement uncertainty introduced by the linearity of the Si photodiode is better than 0.1% (k = 1).

#### 4.2.7. Integrating Sphere Source Non-Uniformity and Stray Light

The standard radiometer and the SVC spectrometer possess similar field-of-view (FOV) angle parameters. During the experiment, their observation regions at the exit port of the integrating sphere largely overlap. The radiance distribution of the integrating sphere source approximates a Lambertian distribution. The measurement uncertainty introduced by the spatial non-uniformity of the integrating sphere radiance is approximately 0.3% (k = 1). The measurement uncertainty introduced by stray light is approximately 0.06% (k = 1) for the standard radiometer and approximately 0.3% (k = 1) for the SVC spectrometer.

#### 4.2.8. Out-of-Band Response of the SVC Spectrometer

The out-of-band response of the SVC spectrometer refers to the response of its spectral channels to radiation at wavelengths outside the passband. In this experiment, the monochromator and the standard lamp-plaque system are used as light sources, respectively. When calibrating the SVC spectrometer with the quasi-monochromatic light from the monochromator, the out-of-band response of the SVC spectrometer itself is rarely excited due to the extremely narrow spectral width of the incident light (on the nanometer scale). Conversely, the standard lamp-plaque covers a wavelength range of 250 nm to 2500 nm, encompassing the entire operational band of the SVC spectrometer during calibration. Consequently, the SVC spectrometer still responds to light outside the passband of the spectral channels, which leads to an underestimation of the radiance responsivity calibrated by the monochromator relative to that calibrated by the standard lamp-plaque.

Experimental verification demonstrates that the out-of-band response of the SVC spectrometer is mainly influenced by light from the short-wavelength direction. Lasers with wavelengths of 473 nm, 532 nm, and 633 nm are utilized as sources to evaluate the out-of-band response of the spectral channels within the 775 nm to 855 nm range. The evaluation indicates that for the spectral channels of the SVC spectrometer in the 775 nm to 855 nm range, the measurement uncertainty introduced by the out-of-band response is approximately 0.7% (k = 1).

#### 4.2.9. Integration Time of the SVC Spectrometer

The DN values of the SVC spectrometer used to calculate the radiance responsivity are integration-time-normalized DN values. Discrepancies exist between the integration times used by the SVC spectrometer in the standard lamp-plaque calibration experiment and the monochromator calibration experiment, with a maximum difference of approximately a factor of 7. Under the same illumination source, the variation in the integration-time-normalized DN of the SVC spectrometer caused by different integration times is approximately 0.5% (k = 1).

Based on the detailed analysis of the individual components discussed above, the comprehensive measurement uncertainty of the monochromator-based radiance calibration system is established. It should be noted that the uncertainty of the primary reference standard (i.e., the standard radiometer) is directly derived from the evaluated uncertainty provided by the NIM. Conversely, all other system-specific components were rigorously quantified through our independent experimental evaluations.

Because all these evaluated components are mutually independent and uncorrelated, they are aggregated using the Root Sum Square (RSS) method [23]. Table 3 summarizes the detailed allocation and proportional contribution of each uncertainty component, yielding a combined standard measurement uncertainty of 1.27% (k = 1) for our proposed monochromator-based calibration system.

### 4.3. Calibration Consistency Analysis

We define the radiance responsivity ratio, ${Ratio=\;R}_{mono}/R_{lamp}$, to evaluate the consistency between the two calibration schemes [24]. Within the experimental spectral range of 775 nm to 855 nm, the uncertainties of the SVC spectrometer’s radiance responsivity calibrated by the monochromator system and the standard lamp-plaque system are 1.27% (k = 1) and 2.55% (k = 1), respectively. The relative combined standard uncertainty of the two calibration systems is calculated to be U_c_ = 2.85% (k = 1). By applying a coverage factor of k = 2 (corresponding to a 95% confidence level), the expanded measurement uncertainty is determined to be U = 5.70%.

**Figure 8:**
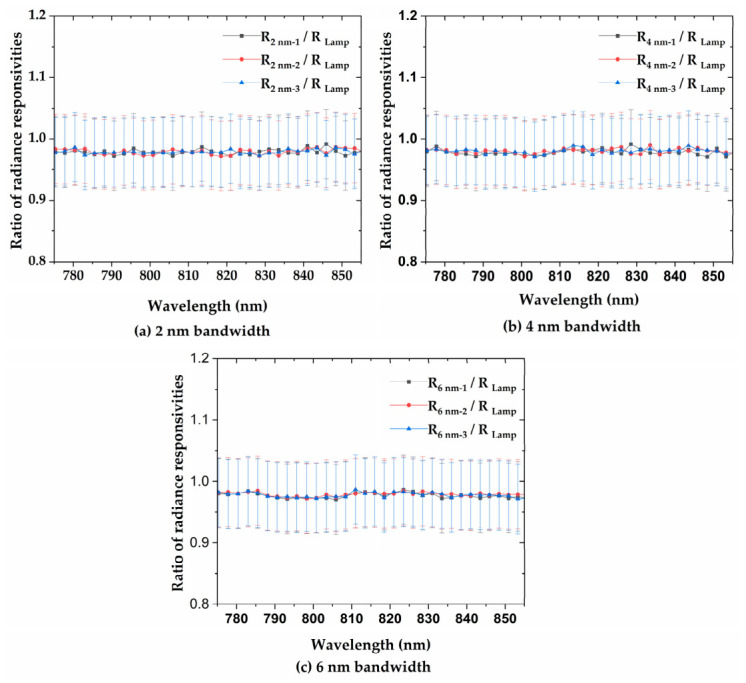
Ratio between the radiance responsivity of the SVC spectrometer as calibrated with the monochromator under varying bandwidths (2 nm, 4 nm, and 6 nm) and the standard lamp-plaque system. The error bars represent the k = 2 expanded combined measurement uncertainty.

Experimental results demonstrate that under the 2 nm, 4 nm, and 6 nm bandwidth configurations, the minimum Ratio is 0.9706, which corresponds to a maximum relative deviation of approximately 2.94%. As shown in Figure 8, when superimposing the k = 2 expanded uncertainty bounds (1.000 ± 0.0570) as error bars around the ideal consistency ratio of 1.000, all measured responsivity ratios across the entire wavelength range fall completely within this uncertainty interval. This result conclusively indicates that the radiance responsivity calibration results from both systems agree well within the combined measurement uncertainty.

## 5. Conclusions

In this study, a systematic cross-verification between a monochromator-based radiance calibration system and a standard lamp-plaque system was conducted through both theoretical derivation and experimental testing, utilizing an SI-traceable standard lamp-plaque system as an independent benchmark.

Starting from fundamental physical principles, an equivalent calibration model for the monochromator-based radiance calibration architecture was established. Theoretical analysis and rigorous numerical integration confirm that the calibration deviation induced by the spectral mismatch between the monochromator’s equivalent synthesized spectral distribution $L_{\sum }\left(\lambda \right)$ and the standard lamp spectrum $\frac{L_{\mathrm{lamp}}\left(\lambda \right)}{L_{\mathrm{lamp}}(\lambda_{0})}$ is approximately 0.31%, which is mathematically insufficient to account for the 2.3% total experimental deviation observed. This study rigorously proves that the deviation is not derived from theoretical spectral mismatch or bandwidth expansion. Instead, it is unequivocally governed by systematic hardware factors: primarily the certified baseline uncertainty of the standard lamp-plaque, the out-of-band stray light response of the SVC spectrometer, and integration time variations. The consistency evaluation, based on the radiance responsivity ratio and expanded measurement uncertainty, confirms that both calibration methodologies agree within the combined measurement uncertainty.

It is important to acknowledge that the empirical validation presented in this study is strictly limited to the 775 nm–855 nm near-infrared band. While the underlying physical convolution mechanism is theoretically valid across a broader range, framing these findings as a general engineering recommendation for the full 380 nm–2500 nm LIBRA spectral range requires caution. Practical extension of this calibration strategy to extreme spectral edges (e.g., UV < 400 nm or SWIR > 2000 nm) involves wavelength-dependent hardware limitations, such as degraded signal-to-noise ratios and intensified stray light characteristics, which were not experimentally addressed in this work. Therefore, while this study provides a crucial empirical basis for the 775 nm–855 nm band, comprehensive full-spectrum (380 nm–2500 nm) validations are planned for the subsequent engineering phases of the LIBRA project to fully assess these wavelength-dependent constraints. By ruling out bandwidth variations as the primary calibration bottleneck, this work provides a solid theoretical and empirical basis for safely adopting wider bandwidths, thereby advancing the measurement precision of the Earth–Moon Imaging Spectrometer toward the ultimate <1% target.

## Figures and Tables

**Table 1:** Relative deviation between the radiance responsivities of the 833.5 nm channel calibrated by the monochromator under different bandwidths and the standard lamp-plaque.

${\Delta \lambda }_{i}$ (nm)	($R_{{\Delta \lambda }_{i}}-R_{\mathrm{lamp}}$)/$R_{\mathrm{lamp}}$
2	−0.31%
4	−0.31%
6	−0.31%

**Table 2:** Relative deviation in the radiance responsivity of the standard radiometer between laser and quasi-monochromatic light measurements.

**Bandwidth (nm)**	$R_{\mathrm{SP}}$ $\left(\mathrm{DN}/W/m^{2}/\mathrm{sr}\right)$	$R_{\Delta \lambda }$ $\left(\mathrm{DN}/W/m^{2}/\mathrm{sr}\right)$	${(R_{\Delta \lambda }\;-\;R}_{\mathrm{SP}})/R_{\mathrm{SP}}$
2	5.2231 × 10^−8^	5.2237 × 10^−8^	0.01%
4		5.2242 × 10^−8^	0.02%
6		5.2254 × 10^−8^	0.04%

**Table 3:** Measurement uncertainty evaluation for the SVC spectrometer radiance responsivity calibration via the monochromator-based radiance calibration system.

Source of Uncertainty	Uncertainty Contribution (%) (k = 1)
Standard radiometer measurement repeatability	0.4
Standard radiometer radiance responsivity	0.32
SVC spectrometer measurement repeatability	0.4
Numerical integration	0.5
Temperature	0.1
Detector response linearity	0.1
Integrating sphere source uniformity	0.3
Stray light	0.3
Out-of-band response of SVC spectrometer	0.7
Integration time of SVC spectrometer	0.5
Electrometer	0.1
Combined uncertainty	1.27

## Data Availability

The original contributions presented in this study are included in the article. Further inquiries can be directed to the corresponding author.
